# Cardiac arrest associated with pneumorrhachis and pneumocephalus after epidural analgesia: two case reports

**DOI:** 10.1186/s13256-018-1908-4

**Published:** 2018-12-22

**Authors:** Hyungoo Shin, Hyuk Joong Choi, Changsun Kim, Inhye Lee, Jaehoon Oh, Byuk Sung Ko

**Affiliations:** 1 Department of Emergency Medicine, College of Medicine, Hanyang University Guri Hospital, 153 Gyeongchun-ro, Guri-si, Gyeonggi-do 11923 Republic of Korea; 2 Department of Emergency Medicine, Hanyang University Seoul Hospital, Seoul, Republic of Korea

**Keywords:** Pneumorrhachis, Pneumocephalus, Cardiopulmonary resuscitation, Epidural analgesia

## Abstract

**Background:**

Epidural analgesia has become a common procedure to provide excellent pain relief with few complications. Pneumorrhachis and pneumocephalus are rare complications of unintentional dural puncture and injection of air into the subarachnoid or subdural space. No cases of cardiac arrest associated with these complications have been reported in the literature previously.

**Case presentation:**

We report cases of pneumorrhachis and pneumocephalus in two Korean women who previously visited a local pain clinic and underwent epidural analgesia. Thereafter, they were admitted to the emergency department with cardiac arrest. Cardiopulmonary resuscitation was performed on these patients, and return of spontaneous circulation was achieved. The brain and spine computed tomographic scans showed pneumorrhachis and pneumocephalus, respectively. These cases demonstrate that pneumorrhachis and pneumocephalus may occur after epidural analgesia, which may be associated with cardiac arrest in patients.

**Conclusions:**

If cardiac arrest occurs after epidural analgesia, pneumocephalus and pneumorrhachis should be considered as its cause. Although epidural analgesia is a common procedure, caution is warranted during this procedure.

## Background

Epidural analgesia is commonly used to provide excellent pain relief with few complications [1]. Although it has become a routine procedure, the complications that are associated with its use may be underestimated [2]. Identification of the epidural space through loss of resistance to air is a widely applied technique during epidural catheter placement [3]. However, several complications are associated with this method, including pneumocephalus [3], subcutaneous emphysema [4], venous air embolism [5], and spinal cord and nerve root compression [6]. Pneumorrhachis and pneumocephalus are rare complications of unintentional dural puncture and air injection into the subarachnoid or subdural space [7]. To the best of our knowledge, no cases of cardiac arrest associated with these complications have been reported. In this report, we describe two cases of cardiac arrest associated with pneumorrhachis and pneumocephalus after epidural analgesia that originated from the use of air during loss-of-resistance placement technique.

## Case presentation

### Case 1

**Fig. 1:**
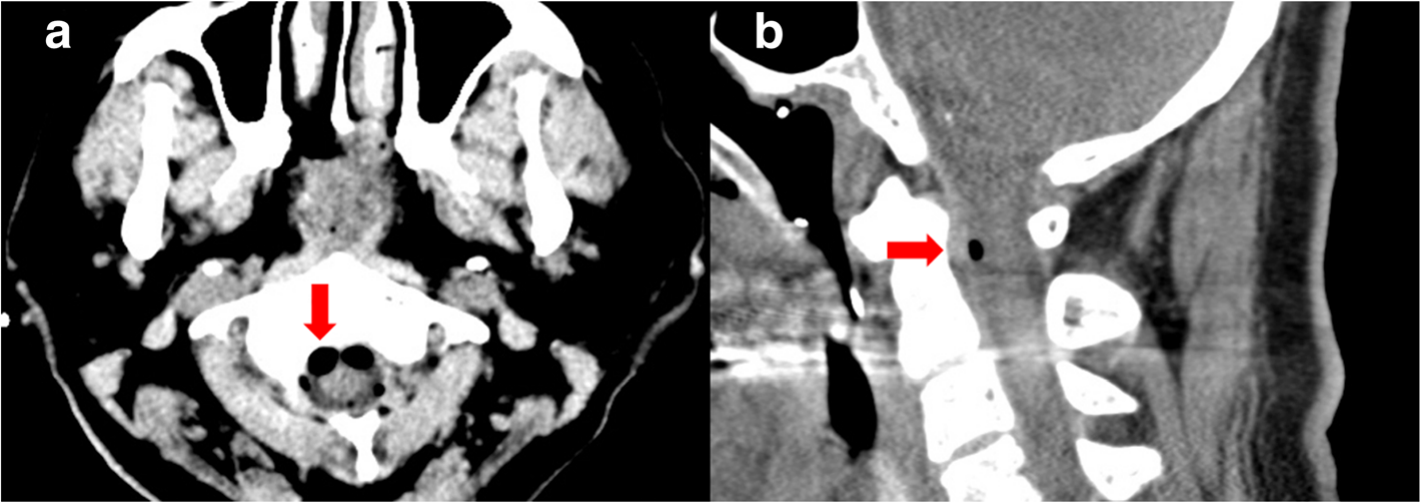
Brain scan showing air (red arrow) in the spinal canal and prepontine cistern (**a**). Spinal computed tomographic scan showing intradural free air (red arrow) at the C3 level (**b**)

Two patients presented to an emergency department (ED) with cardiac arrest (Tables 1 and 2). The first patient was a 78-year-old Korean housewife with a medical history of hypertension. She had no social or environmental risk factors and no family history of cardiac disease. She visited a local pain clinic and underwent epidural analgesia for back pain control 59 min before going to the ED. Her blood pressure decreased as she lost consciousness. Cardiac arrest occurred 20 min after the procedure. The patient’s initial rhythm was asystolic. The emergency medical services team performed cardiopulmonary resuscitation (CPR) on the patient during transport to the hospital. She arrived at the ED with asystole and no measurable vital signs. Her Glasgow Coma Scale (GCS) score was 3 points. Her pupils were fully dilated, and all of her brainstem reflexes were lost. Successful return of spontaneous circulation (ROSC) was achieved after 4 min of CPR. Her blood pressure was 105/33 mmHg, and her heart rate was 79 beats per minute. Spinal computed tomography (CT) performed after ROSC showed air in the spinal canal and prepontine cistern and intradural free air at the C3 level (Fig. 1). The patient was admitted to the intensive care unit (ICU), and post-cardiac arrest care interventions were performed. On the patient’s fifth day of hospitalization, she underwent diffusion-weighted magnetic resonance imaging, which showed diffuse signal changes in the bilateral frontotemporoparietal cortex, basal ganglia, and hippocampus, suggesting hypoxic brain injury. Electroencephalography demonstrated diffuse cerebral dysfunction. On the tenth day of hospitalization, she was transferred to a long-term care facility with Cerebral Performance Categories scale (CPC) 4 status. She died 11 days after leaving the hospital.

**Table 1:** Patient characteristics

	Case 1	Case 2
Sex	Female	Female
Age, years	78	69
Past medical history	Hypertension	None
Allergic history	Unknown	Unknown
Smoking/alcohol	None	None
Injection drug	Triamcinolone 40 mg with lidocaine 60 mg	Triamcinolone 40 mg with lidocaine 60 mg
Type of procedure	Epidural analgesia	Epidural analgesia
Time interval (min)		
From injection to cardiac arrest	20	35
From cardiac arrest to visiting ED	39	39
CPR duration	4	6

*Abbreviations*: *CPR* Cardiopulmonary resuscitation, *ED* Emergency department

**Table 2:** Laboratory and radiologic findings after return of spontaneous circulation

	Case 1	Case 2
ABGA		
pH	7.18	7.05
pCO_2_ (mmHg)	55.9	51.1
pO_2_ (mmHg)	48.2	99.2
HCO_3_^−^ (mmol/L)	21.0	14.2
Base excess (mmol/L)	−6.9	−15.6
Lactate (mmol/L)	5.6	13.4
WBC (/mm^3^)	8,700	15,700
Hb (g/dl)	11.2	11.1
Hct (%)	34.4	35.3
PLT (/mm^3^)	175,000	175,000
Serum Na^+^/K^+^/Cl^−^ (mEq/L)	140/4.6/107	139/3.2/106
Glucose (mg/dl)	140	516
BUN/creatinine (mg/dl)	18.1/0.75	16.0/0.9
AST/ALT (mg/dl)	24/27	41/26
CRP (mg/dl)	< 0.3	< 0.1
Cardiac troponin I (ng/ml)	< 0.05	< 0.05
NSE (ng/ml)	22.1 (After ROSC)	Not measured
	59.8 (After 24 h from ROSC)	
	163.0 (After 48 h from ROSC)	
RUA	Nonspecific finding	Nonspecific finding
Chest x-ray	Diffuse bronchovascular bundle thickening in both lungs	Cardiomegaly with pulmonary congestion
Electrocardiography	Sinus tachycardia	Sinus tachycardia
Echocardiography	No RWMA	No RWMA
Brain CT	Air in the spinal canal and prepontine cistern	Extensive anoxic brain damage and extensive pneumocephalus
Electroencephalography	Diffuse cerebral dysfunction	Diffuse cerebral dysfunction
DW-MRI	Hypoxic brain injury	Hypoxic brain injury

*Abbreviations*: *ABGA* Arterial blood gas analysis, *AST* Aspartate aminotransferase, *ALT* Alanine aminotransferase, *BUN* Blood urea nitrogen, *CT* Computed tomography, *CRP* C-reactive protein, *DW-MRI* Diffusion-weighted magnetic resonance imaging, *Hb* Hemoglobin, *Hct* Hematocrit, *PLT* Platelets, *NSE* Neuron-specific enolase, *ROSC* Return of spontaneous circulation, *RUA* Routine urinalysis, *RWMA* Regional wall motion abnormality, *WBC* White blood cell

### Case 2

**Fig. 2:**
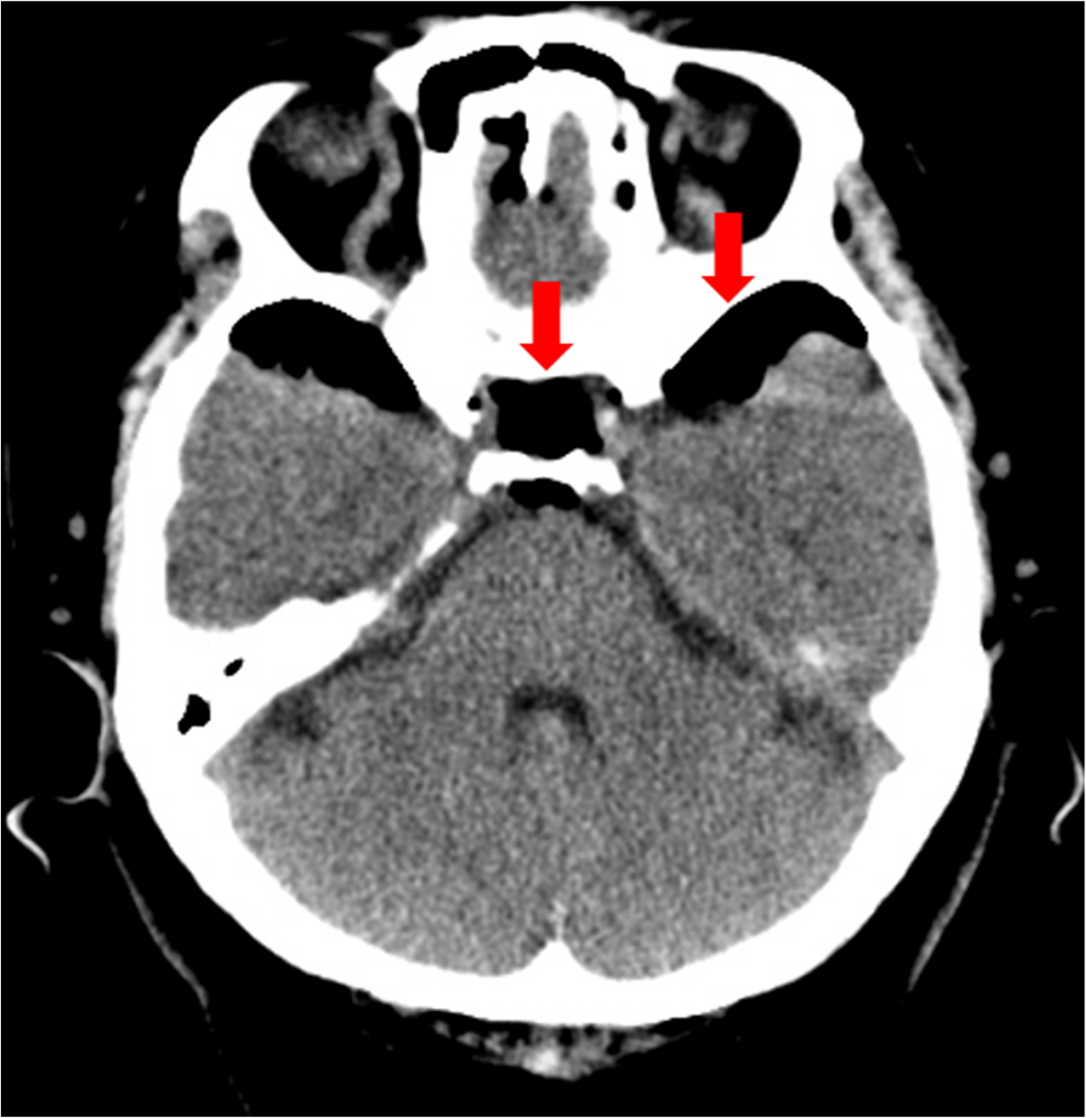
Brain computed tomographic scan demonstrating extensive anoxic brain damage and extensive pneumocephalus (red arrow), bilateral middle and lower frontal convexity and Sylvian fissures

A 69-year-old Korean housewife presented to the ED with cardiac arrest. She had no past medical, social, or environmental risk factors and no family history of cardiac disease. She visited a local pain clinic and underwent epidural analgesia 74 min before visiting the ED. She lost consciousness, and cardiac arrest occurred 35 min after the procedure. She had initial asystole. The emergency medical services team performed CPR and transported her to the ED. She arrived at the ED with asystole and no measurable vital signs. Her GCS score was 3 points. Her pupils were fully dilated, and all brainstem reflexes were lost. Successful ROSC was achieved after 6 min of CPR. Her blood pressure was 225/150 mmHg, and her heart rate was 104 beats per minute. Brain CT after ROSC demonstrated extensive anoxic brain damage and pneumocephalus, bilateral middle and lower frontal convexity, and Sylvian fissures (Fig. 2). The patient was admitted to the ICU, and post-cardiac arrest care interventions were performed. Her metabolic acidosis progressed, and she underwent continuous renal replacement therapy for 15 days. Electroencephalography showed diffuse cerebral dysfunction. On the 31st day of hospitalization, she was transferred to a long-term care facility with CPC 4 status. She died 14 days after leaving the hospital.

## Discussion and conclusions

We report two cases of pneumorrhachis and pneumocephalus in patients who previously underwent epidural analgesia. Thereafter, they presented to the ED with cardiac arrest. To the best of our knowledge, no cases of cardiac arrest associated with these complications have been reported previously.

Pneumorrhachis is a phenomenon characterized by intraspinal air mostly due to traumatic and iatrogenic etiologies [8]. Various conditions may cause pneumorrhachis, including iatrogenic manipulations during interventions and lumbar puncture [9]. The diagnostic tool of choice for the detection of pneumorrhachis is CT [10]. Pneumorrhachis is usually asymptomatic and reabsorbs spontaneously and completely the air that passed directly into the blood for several days without recurrence [11]. Therefore, patients with pneumorrhachis are commonly managed conservatively.

The symptoms of pneumocephalus, such as headache, elevated intracranial pressure, vomiting, convulsions, and unstable vital signs, depend on the intracranial air distribution and amount [12]. Less than 2 ml of subarachnoid air can cause headache [13]. However, the air volume that can safely be injected into the epidural space remains to be established [14], and the correlation between the intracranial air amount and headache is unclear [13].

The entrapped air occupies parts of the cerebrospinal compartment, which may cause both intracranial and intraspinal hypertension and hypotension secondary to either increase or decrease in intracranial and intraspinal pressure [8]. The injected air can also act as a space-occupying lesion and exert pressure on the nervous structures within the spinal canal. Hence, entrapped intraspinal air might cause tension pneumorrhachis and pneumocephalus with nervous tissue compression [15]. These mechanisms may lead to cardiac arrest. Therefore, recognizing the differential diagnosis of altered intraspinal pressure within the cerebrospinal compartment is important to ensuring its adequate management.

There are some limitations to these conclusions. There are other causes of cardiac arrest that were not thoroughly investigated in these cases. Electrocardiograms revealed sinus tachycardia, and the cardiac troponin levels were not elevated in these cases of cardiac arrest. Furthermore, the echocardiograms of the patients displayed no regional wall motion abnormality. However, the exclusion of cardiac causes had not been achieved through coronary angiography. Additionally, anaphylactic shock due to the injected anesthetic drug may have resulted in cardiac arrest. However, we believe that fatal anaphylaxis may be distinguished from pneumorrhachis in that the former can occur within minutes of drug injection compared with the latter. The possibility of total spinal anesthesia following epidural analgesia also needs to be ruled out. Total spinal anesthesia causes sudden physiological changes by blocking the peripheral nerves, including the spinal cord and cranial nerve [16]. Because both patients came from local pain clinics, there was insufficient information to investigate this possibility; including the patients’ position, location of injection, type or size of needle, and number of attempts. Thus, it is difficult to further clarify the association between total spinal anesthesia and cardiac arrest. In some case reports, pneumorrhachis or pneumocephalus developed after basic life support, and this possibility cannot be excluded [17].

No empiric treatment guidelines or standards of care exist for pneumorrhachis, owing to its rareness and different pathogenesis and etiologies. Pneumorrhachis is thought to be associated with increased morbidity and mortality [8]. In this report, we present cases of pneumorrhachis and pneumocephalus in two women who presented to the ED with cardiac arrest after epidural analgesia. If cardiac arrest occurs after epidural analgesia, pneumocephalus and pneumorrhachis should be suspected as its cause. The contributing factors for pneumorrhachis and pneumocephalus have to be evaluated, and appropriate interventions should be implemented. Patients with severe and life-threatening conditions that can lead to pneumorrhachis and pneumocephalus should be carefully monitored, followed, and considered for admission to an ICU. Care should be taken because patients may have a cardiac arrest after epidural analgesia.

## Abbreviations

ABGA: Arterial blood gas analysis
ALT: Alanine aminotransferase
AST: Aspartate aminotransferase
BUN: Blood urea nitrogen
CPC: Cerebral Performance Categories scale
CPR: Cardiopulmonary resuscitation
CRP: C-reactive protein
CT: Computed tomography
DW-MRI: Diffusion-weighted magnetic resonance imaging
ED: Emergency department
GCS: Glasgow Coma Scale
Hb: Hemoglobin
Hct: Hematocrit
ICU: Intensive care unit
MRI: Magnetic resonance imaging
NSE: Neuron-specific enolase
PLT: Platelets
ROSC: Return of spontaneous circulation
RUA: Routine urinalysis
RWMA: Regional wall motion abnormality
WBC: White blood cell
